# Supporting cross-cultural online discussion with formal and informal platforms: a case between Hong Kong and Taiwan

**DOI:** 10.1186/s41039-017-0050-z

**Published:** 2017-01-23

**Authors:** Liping Deng, Yang-Hsueh Chen, Sandy C. Li

**Affiliations:** 1 Department of Education Studies, Hong Kong Baptist University, AAB 834 Kowloon Tong, Hong Kong; 2 Department of Education, National University of Tainan, Tainan, Taiwan, ROC

**Keywords:** Cross-cultural communication, Online discussion, Course management system, Facebook, Higher education

## Abstract

The paper reports on a collaborative project on fostering cross-cultural online discussion between two universities with one in Hong Kong and the other in Taiwan. Two online platforms—Moodle as a formal channel for course-related discussion and Facebook as an informal channel for social-oriented interaction—were employed. The study pays special attention to instructional design based on research-derived strategies and reflects on the effects of various strategies as well as students’ experiences and perceptions. The results show that the students perceived the online discussion as valuable for exchanging perspectives and enhancing cross-cultural understanding. However, they were less motivated to use Facebook as the informal channel for socialization. The challenges encountered especially those concerning using Facebook in formal learning environments will be discussed. At the end, the recommendations for better utilizing and bringing together formal and informal online platforms will be suggested.

## Introduction

With growing pervasiveness and ubiquity in our society, web-based technologies have been widely used to compliment traditional face-to-face teaching. The resulting blended mode of learning is regarded as a preferred and viable option (Harrington, Gordon, & Schibik, 2004) as it brings together the strength of online and face-to-face learning (Garrison & Vaughan, 2008). Moreover, online platforms also provide an affordable and accessible means for cross-cultural communication by connecting students from different countries (Commander, Zhao, Gallagher, & You, 2016). It is expected that a culturally diverse group will bring forth multiple perspectives, which will contribute to the development of reflective thinking and cross-cultural understanding (Häkkinen & Järvelä, 2006; Shadiev, Hwang, & Huang, 2015). When looking outside of classrooms, social networking websites (SNS) (e.g., Facebook, Twitter, Google+) have become increasingly pervasive in student daily life, which gives rise to a growing interest in the educational potential of SNS. Still, the pedagogical values of Facebook in formal learning domain remain inconclusive, and studies have reported a mixed attitude and views of using Facebook for learning purposes among students (Manca & Ranieri, 2013). On the whole, empirical work that focuses on designing and implementing cross-cultural collaborative learning is still limited. Existing studies on the theme usually involve a single online platform, be it formal like Moodle or Blackboard (e.g., Yang, Kinshuk, Yu, Chen, & Huang, 2014), or informal like Facebook (e.g., Wang, 2011). There have not been studies, to the best of our knowledge, which bring together both formal and informal learning platforms in support of cross-cultural collaboration.

The current study seeks to connect students from two universities in Asian-pacific regions (Hong Kong and Taiwan) and engage them in online discussions via two online platforms—Moodle as a formal channel for course-related discussion and Facebook as an informal channel for social-oriented interactions. The study accentuates on students’ perspectives and attitudes towards the use of both formal and informal online platforms. It seeks to answer two research questions: (1) What are the students’ experience and perceptions of cross-cultural online discussion? (2) What are the students’ experience and perceptions of using Facebook as an informal channel for communication? The results of our study can provide valuable insights into students’ perspectives of cross-cultural online discussion, the application of both formal and informal channels of communication, and strategies to leverage them for educational purposes.

### Review of literature

A computer-supported learning environment, as Kreijns, Kirschner, and Jochems (2003) denoted, consisted of both cognitive and socio-emotional processes. Yet unfortunately, the socio-emotional processes that concerned with “getting to know each other, committing to social relationships, developing trust and belonging, and building a sense of on-line community” (p. 342) are often neglected or forgotten in formal instructional settings. Adapted from Kreijns, Kirschner, and Vermeulen (2013), Fig. 1 shows a framework of formal/informal learning that helps to conceptualize our study and structure literature review (we will refer back to this figure when it comes to instructional design later on). It situates learning within formal and informal contexts and recognizes cognitive and socio-emotional dimensions associated with learning experiences. In light of this, the current study seeks to bring together technological platforms from formal and informal contexts in support of both cognitive and social dimensions of learning. The following literature review will first look into the use of online discussion to enhance learning within formal learning contexts. Next, we will present related studies on cross-cultural communication via online platforms. Lastly, a body of scholarly work on the use of Facebook, a popular SNS, for educational purposes will be put into perspective.

**Fig. 1:**
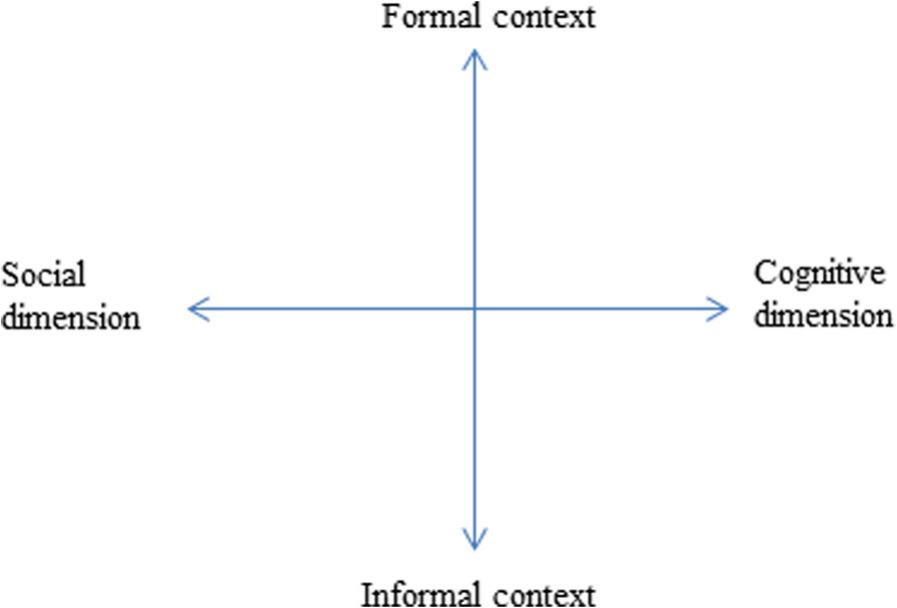
Framework of formal/informal learning

### Online discussion as pedagogical tool

As web-based technologies become more and more pervasive and ubiquitous, they have been increasingly used to support diverse forms of learning. Within the formal context, course management systems (CMS) have been mainstreamed in the tertiary institutions globally (Coates, James, & Baldwin, 2005). The educational values of asynchronous online discussion, usually a standard feature of CMS, have been well documented. Asynchronous online discussion, defined as “a text-based computer-mediated communication environment that allows individuals to interact with one another without the constraint of time and place” (Hew, Cheung, & Ng, 2010, p. 572) has been well accepted as a powerful tool to foster student critical thinking, collaboration, knowledge construction, and cross-cultural understanding (e.g., Barnett-Queen, Blair, & Merrick, 2005; Jorczak & Dupuis, 2014; Chang, Chen, & Hsu, 2011). When participating in online asynchronous discussion, students can have sufficient time to think, reflect, search for relevant information (Lee, 2012), and articulate their ideas in more thought-out and structured ways (Merryfield 2003; Tiene, 2000).

Acknowledging its educational value, researchers continue to probe into the motivating as well as inhibiting factors that influence students’ participation in online discussion. For example, Cheung, Hew, and Ng (2008) identified relationship with peers and knowledge about topics as two vital determinants of student online contributions. Xie, Debacker, and Ferguson (2006) pinpointed five themes that influenced university students’ participation in online discussion including instructor’s role, interaction among peers, discussion topics, course requirements, and usability of online system. Through a comprehensive review of 50 empirical studies, Hew, Cheung, and Ng (2010) summarized several barriers for online discussion including lack of felt need, behavior of other participants (e.g., lack of response), student personality traits (e.g., lack of curiosity and openness), difficulty in keeping up with the discussion or deciding on what to contribute, and technical aspects (e.g., usability issues). Without doubt, careful consideration of instructional design elements, such as topic relevancy/difficulty, student and instructor roles, and facilitation styles, is critical to ensure interactive and mindful learning via online discussion.

### Cross-cultural collaborative learning

Quite a number of empirical studies have been conducted on enhancing cross-cultural communication with the support of digital technologies. Commander and colleagues (2016) offered evidence of collaborative knowledge construction through cross-national online discussion. Similarly, Shadiev, Hwang, and Huang (2015) had students from different countries which play cultural-themed games and communicate through asynchronous and synchronous platforms online. The results showed positive effects of the online activities on enhancing participants’ cross-cultural understanding.

Merryfield’s (2003) study helped to shed lights on the positive impact of online asynchronous discussion by taking a close look at how graduate students interacted with people from other countries through online forums. It was revealed that students could think more deeply about the content and become more focused on what they write, thus contribute to more meaningful and in-depth online discussion. In a way, online platform acted as a “veil to protect people as they reveal, question, and take risks” (Merryfield, 2003, p. 156), thus encouraged equity in participation since shy or those who were less competent with writing could take time to think, react, and participate.

However, some challenges have been noted and endeavors have been made to ferret out the effective strategies for cross-cultural collaborative learning. Kim and Bonk (2002) compared the online collaborative behaviors of student teachers from three different cultures and pointed out language as a barrier for some students’ participation in online discussion. Yang, Kinshuk, Yu, Chen, and Huang (2014) sought to identify the effective strategies for cross-cultural collaborative learning by bringing the university students in the USA and China together in online discussion. They identified the social interaction and cultural interaction as the cornerstone for cross-cultural collaboration and recommended a series of topics for online discussion including social lounge, cultural orientation, and technology integration. Similarly, Wang (2011) also focused on the instructional design issues of cross-cultural collaborative projects, in specific, concerning group size and task types (formal vs. informal tasks). The study showed that small group size with two to three students were preferred since it allowed for more chances of interaction.

### Facebook for educational purposes

In students’ life outside school, social media especially SNSs have penetrated their daily and social lives. Some studies have shown students’ preferences of SNS over CMS. For instance, Schroeder and Greenbowe (2009) noted that undergraduate students were in favor of using Facebook for online discussions instead of CMS provided by the university. DiVall and Kirwin (2012) reported that students were more active in posting on Facebook than on traditional CMS. Similarly, Gray, Annabell, and Kennedy (2010) found medical students were drawn to Facebook instead of CMS at their university for group study. Deng and Tavares (2013) also observed that pre-service teachers resorted to their own Facebook group for peer interaction and support showing little interest in the formal discussion forum on CMS. Within the cross-cultural settings, Wang’s (2011) study recognized Facebook as a valuable platform for online collaboration. McCarthy (2010) denoted that Facebook could help mitigate the language barriers and social inhibitions in the interaction among local and international students in an Australian university.

However, Manca and Ranieri (2013), through a critical review of the educational uses of Facebook, espoused that its educational value was still questionable. Madge, Meek, Wellens, and Hooley (2009) maintained that although university students sometimes interacted with their peers on Facebook about academic matters, they were not keen on using Facebook for formal learning-related discussions. In resonance, Fewkes and McCabe (2012) also reported that although the majority of students admitted that they had used Facebook for educational purposes, it was ranked the lowest among others (e.g., updating status, checking friends’ status). Gray, Annabell, and Kennedy (2010) reported through a survey among medical students in Australia, only a quarter of students acknowledged the use of SNS for supporting learning. It seems that students still looked on Facebook as primarily a platform for social purposes (Madge, Meek, Wellens, & Hooley, 2009) and preferred to have their learning and social spaces separated (Jones, Blackey, Fitzgibbon, & Chew, 2010). That helped explain the negative attitudes towards the use of Facebook for academic purposes especially when teachers initiated it or got involved (Gettman & Cortijo, 2015). Another plausible explanation might be that the traditional educational model rendered a learning experience drastically different from that of the out-of-school experience with Facebook (Crook & Cluley, 2009). As such, Crook and Cluley suggested adopting “a more informal disposition” (p. 202) towards using SNS within formal educational settings.

## Methods

### Participants and contexts

The study involved 75 students from two medium size comprehensive universities—one in Hong Kong and the other in Taiwan. The students at both sides were all education majors at similar year levels who took similar courses concerning ICT for education. There were 59 Hong Kong students participated in the study, and they were year 1 and year 2 students taught by two instructors (class A with instructor A; class B with instructor B). The involved instructors were the researchers of the present study. For the Taiwan institute, the participating class consisted of 16 students who were all year 2 students.

### Instructional design

**Fig. 2:**
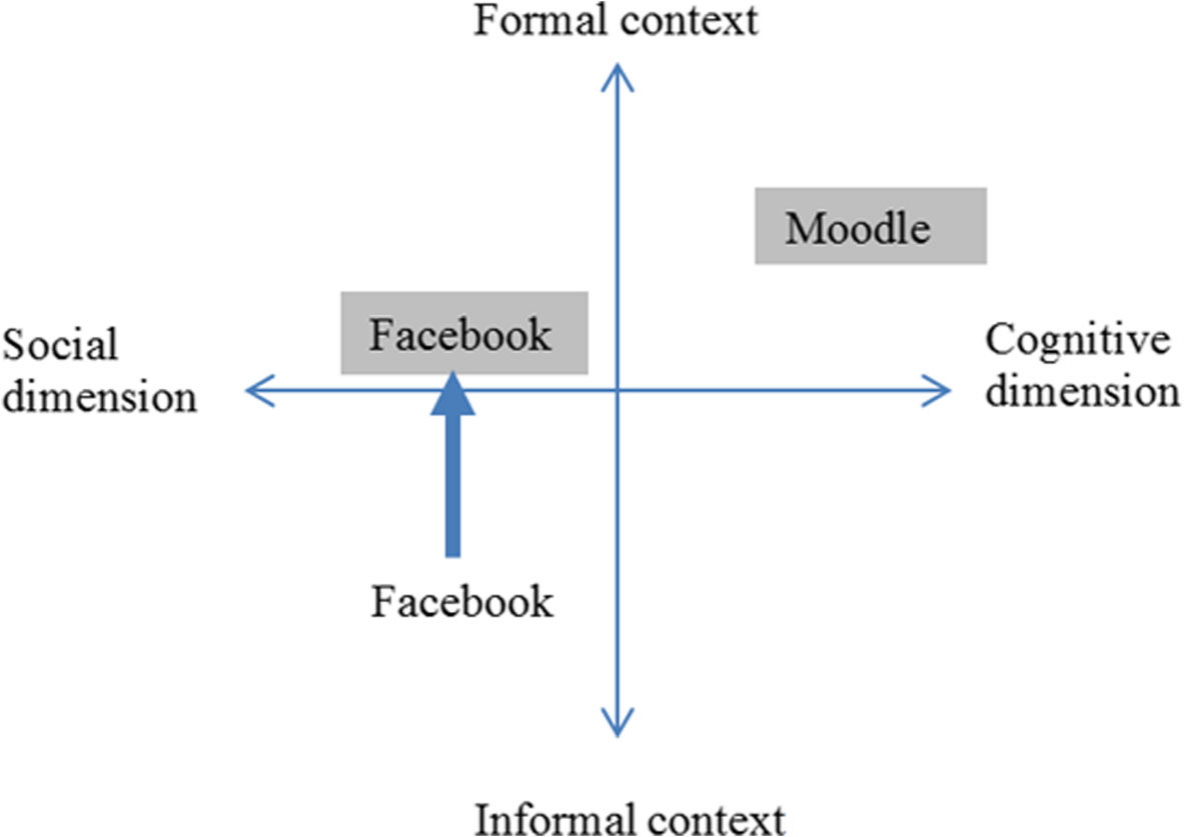
Instructional design of cross-cultural discussion

Three instructors (two from Hong Kong and one from Taiwan) worked closely in the instructional design which was greatly informed by related literature concerning online discussion and community (e.g., Deng & Yuen, 2011; Kreijns, Kirschner, & Jochems, 2003; Preece, 2000). Skype served as the main tool for many rounds of discussion over a wide range of issues from purpose, technological tools, grouping, as to logistic issues such as time, account creation, and technical support. Two online platforms—Moodle as a formal channel for cognitive purposes and Facebook as an informal channel for social purposes—were selected to support the online discussion between Hong Kong and Taiwan students. Such a design was rooted in the beliefs that (1) a learning community consisted of cognitive and social dimensions (Kreijns, Kirschner, & Vermeulen, 2013) and (2) social presence and interactions can contribute to conducive climate for learning (Crook & Cluley, 2009; Deng & Yuen, 2011). One of the research agenda was to explore whether Facebook, the most popular SNS in the informal domain, could make its way into a more formal educational setting and complement a formal system like Moodle (as shown in Fig. 2). Another thing worth pointing out is that formal/informal and cognitive/social dimensions should be looked upon as two ends of continua. Thus, Moodle is situated at one end of formal and cognitive dimensions, whereas Facebook is situated on the other end of social dimension yet crosses the border of both formal and informal contexts.

Table 1 lays out some vital design decisions made for using formal and informal online platforms. To begin with, the main purpose of the formal online platform was to engage students in online discussions related to course subjects in order to enhance reflective thinking and cross-cultural understanding. Moodle, the CMS used in the university in Hong Kong, was selected as the platform for formal online discussion. The students from both universities were rearranged into three groups (each contained about 20 Hong Kong students and 5 Taiwan students) out of two main reasons: First, the related literature (e.g., Kim, 2013) showed that students in smaller discussion groups tended to be more engaged and interactive. Second, we intended to maintain cultural diversity and to ensure similar and balanced group compositions. Resonant to Yang et al. (2014)’s recommendation of having cultural-oriented discussion first, the first topic suggested for discussion was “Introducing the education system in Hong Kong and Taiwan.” The second and third topics were closely related to the course content: “Technology for teaching and learning” and “Role of mobile apps for learning,” respectively. For each discussion topic, the instructors first posted general guiding questions and the students were encouraged to respond, initiate new threads under the theme, or respond to each other.

**Table 1:** Instructional design regarding formal and informal platforms

	Formal platform	Informal platform
Technological tools	Moodle	Facebook
Purpose	Course-related online discussion to enhance cross-cultural understanding and reflection on course content	Informal and social-oriented discussion to enhance social interaction and relationships
Grouping	Three smaller culturally mixed groups	One big group with everyone interested in
Topics	General topics set for each discussion forums.	Free discussion
Facilitation	Instructors as facilitators	RAs as facilitators

Furthermore, related literature concerning online participation and discussion facilitation (e.g., Salmon, 2000) was drawn upon to motivate online participation and facilitate online discussion. A small percentage of marks were assigned for online participation. Each student was required to post at least twice every week and advised to post one new post and one response to other’s posts. The guidelines for online discussion (e.g., read before post to avoid redundancy) and tips on writing as conversing (e.g., how to elicit others’ responses) were shared with the students. Additionally, one instructor and one research assistant took the role as online facilitator who monitored online posts, responded, raised questions, prompted for further reflection, and summarized the main points. Another emerging issue concerned the language for communication. For the university in Hong Kong, the medium of instruction was English, while the university in Taiwan used Chinese. To promote participation, the students could choose the language they were comfortable with.

Central to our study is the accentuation of the sociability dimension of learning. As mentioned earlier, due to its popularity in both Hong Kong and Taiwan, Facebook was selected for informal and social-oriented communications which would contribute to the development of social relationships. A Facebook group was set up by one of the instructor as a closed group with memberships granted by invitation only. The students at both sides could choose to join the group. As recommended by many scholars (e.g., Liu, 2007; Shadiev, Hwang, & Huang, 2015; Wang, 2011), self-introduction is a vital initial step for building rapport. The students were suggested to post links of their self-introduction pages created with a sticky note website called *Lino*. After the initial encouragement, the instructors deliberately refrained from showing their presences in the Facebook group for fear to compromise the informal and social atmosphere. Two research assistants based in Hong Kong took the responsibility as the facilitators of the group. Besides Moodle and Facebook, two synchronous online video conferencing sessions (one at the beginning, the other at the end of project) were arranged allowing the students at both sides to meet online. Such face-to-face communication online has been shown to contribute to personalization and motivation in cross-cultural collaborative settings (Kim & Bonk, 2002). 

### Data collection and analysis

They study took an exploratory approach by focusing on students’ experience and perceptions. The sources of data included questionnaires, interviews, and student reflections as part of the course assignments. At the end of the semester, the students were invited to finish an online questionnaire that aimed to understand their experiences and perceptions of the cross-cultural discussion, as well as factors for their online participation such as interest in the topics, responses from peers, comfort level with online discussion. Most questions were in Likert scale format with 1 for strongly disagree and 5 for strongly agree. Also, included in the questionnaire were several open-ended questions that sought to elicit students’ views on (1) perceived values of cross-cultural communication and (2) factors that influenced their engagement with Facebook. Forty-one students have finished the online questionnaires, of which 38 were valid for analysis.

Additionally, several students from both sides were selected and invited to participate in individual interviews. The criteria for selection included students’ levels of online participation, gender, and group membership. We first invited volunteers and then we hand-picked some other students to fulfill the criteria. Eleven students (seven from Hong Kong and four from Taiwan) participated in the individual interviews sessions that lasted for 45 to 60 min. Through the interviews, we probed deeper into students’ experiences and perceptions of various technologies, discussion forums and Facebook in particular, in support of their learning. The interview questions were semi-structured with some customized questions based on the students’ online participation and probing questions based on their interview responses. For instance, for a girl who was quite active in online discussion, the questions asked included What do you see as the major benefits of online discussion? What are the factors that motive you to participate?

As part of the individual assignments, the students wrote personal reflections on the online discussion experiences at the end of the semester. The guiding questions for reflection were “What have you gained from the cross-cultural online discussion? What are the challenges? Can you suggest how to address these challenges?” During data analysis, we used NVivo to pool together and analyze qualitative data including answers to open-ended questions of the questionnaire, interview data, and student reflections. The data from the three sources were analyzed thematically separately; then, the codes were merged at the end.

## Results

### Cross-cultural online discussion on Moodle

As mentioned earlier, there were three discussion folders created on Moodle each for different topics related to ICT and education. The students’ activities on forums, including number of views and posts, were extracted from system logs. Altogether, the students from both institutes posted 301 messages on the three discussion topics (124 for topic 1, 95 for topic 2, and 82 for topic 3) and 263 responses (126 for topic 1, 71 for topic 2, 66 for topic 3). On average, each student posted 3.35 times on topic 1, 2.57 times on topic 2, and 2.21 on topic 3. As the student could choose to post in languages they were comfortable with, the posts on the forums showed the mixture of both English and Chinese.

In the questionnaire, questions were asked to gather students’ perceived benefits of participating in the Moodle forum. As shown in Table 2, the students in general showed positive views of the forum in enhancing communication especially in exchanging ideas (*M* = 4.18, SD = 0.51), enhancing cross-cultural understanding and communication (*M* = 4.05, SD = 0.66), and improving the ability of multi-angle thinking (*M* = 3.97, SD = 4.92). Ninety percent of the students strongly agreed or agreed that they learned new knowledge from other students’ posts (*M* = 4.13, SD = 0.67). Almost all the participants (95%, 36) strongly agreed or agreed that they could organize and present their ideas better in the online discussing platform (*M* = 4.18, SD = 0.51).

**Table 2:** Perceived benefits of participating in the Moodle forum (*n*=38)

Perceived benefits of online discussion	SA/A	*N*	D/SD	*M*	*SD*
Exchanging ideas	36 (94.7%)	2 (5.3%)	0	4.18	.51
Encouraging critical thinking	27 (71.1%)	11 (28.9%)	0	3.76	.54
Improving the ability of multi-angle thinking	33 (86.8%)	5 (13.2%)	0	3.97	.49
Enhancing cross-cultural understanding and communication	33 (86.8%)	4 (10.5%)	1 (2.6%)	4.05	.66
Learn new knowledge from others’ posts	34 (89.5%)	3 (7.9%)	1 (2.6%)	4.13	.67
Organize and present ideas better on online discussing platforms	36 (94.7%)	2 (5.3%)	0	4.18	.51

The qualitative data collected through open-ended questions, interviews, and the reflection assignment also echoed the positive views of online discussions on Moodle. What mentioned most was the excitement of knowing more about people as well as educational contexts and realities at both places. The students described the cross-cultural experience as a “golden opportunity” that helped “extend our horizon.” One student commented that “The students from Hong Kong were very active and their posts were thought-provoking. They also attached the related websites for my reference.” Students also showed the appreciation of the technology that “bound us together” as one commented “I strongly feel the power of Information and Communication Technology.” One Taiwan student remarked that the online sharing experience provided a vivid example of how technology could be integrated into learning, which became a valuable gain for the ICT in Education course.

However, some students perceived the online discussions as too formal and time-consuming. One student commented that “sometimes I found that not many people had time to read essay-length answers.” In resonance, another student recommended adding some less formal topics for discussion in order to maintain people’s interest. Another good suggestion by two students was to assign students to lead the discussion topics. As mentioned by a student, “Responsibility can motivate me more.” As mentioned earlier, Hong Kong students could write in either English or Chinese on Moodle. Yet, one Hong Kong student observed that “Taiwan students are not eager to respond to posts written in English.” From the Taiwan side, the students showed different reactions to the language issue: some felt overwhelmed by the English posts while others appreciated the opportunity to communicate with Hong Kong students in English—they regarded it as a good chance to improve their language ability. Table 3 provides a summary of the main themes concerning students’ perceived benefits and constraints of Moodle and Facebook.

**Table 3:** Summary of the main themes from interview data

	Benefits	Constraints
Moodle	•Cross-cultural sharing •Use of online technologies	•Online discussion is time-consuming •Language issues
Facebook	•Self-introduction as a good start	•Unfamiliarity •Lack of focus •Group is too big •Time constraint •Unclear purposes •Voluntary participation

### Students’ activities on and perception of Facebook

Both groups of students were regular users of Facebook. In the questionnaire, they rated their frequency of visiting Facebook on a five-point scale (5 = “frequently”; 1 = “never”) as 4.11 on average. When probing into how often they used Facebook for learning-related activities on the same frequency scale, it turned out that the students used it quite often for doing school-related work (e.g., group projects) (*M* = 3.37, SD = .91). But they were not active in asking study-related questions (*M* = 2.92, SD = .94) or sharing study-related news/articles (*M* = 2.79, SD = 1.02) on Facebook.

**Fig. 3:**
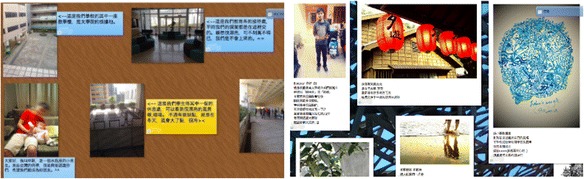
Students’ self-introduction pages designed with Lino

Altogether, 39 students joined the Facebook group and 32 of them participated in the suggested activity of posting self-introductions there. As shown in Fig. 3, the students posted pictures and texts on Lino to introduce their school lives and hobbies. After the initial self-introduction, the group did not see much interaction. One of the instructors from Hong Kong created the Facebook group and only posted the welcome message at the beginning. The two research assistants tried several ways to enhance social presence and interactions within the group, for example, like students’ postings and launching informal topics related to students’ life. Unfortunately, the efforts did not stimulate much interest. Towards the end of the courses, the students were encouraged to upload their group presentation to the group to share with the students on the other side. Two Taiwan groups and one Hong Kong group uploaded their work.

During the interviews, the students generally agreed that the use of Facebook to supplement Moodle discussion was “a good idea.” They also acknowledged Facebook as a potentially valuable platform for socializing and informal communication. Particularly, many expressed that the Facebook sharing of the Lino self-introduction page was a vital first step towards getting to know each other. One commented it was like “opening a window” into the life of other people.

On the other hand, the students also shared their reasons for passive participation in the Facebook group, among which the most salient was *unfamiliarity among participants*. As mentioned by the students, they usually formed a Facebook group with someone they knew face-to-face or people who shared same interests. Since “we don’t know each other well,” the students felt it very hard to “find the topic of conversation.” The design decision of having one big Facebook group further exacerbated these problems. As one student noted, “there are too many people in the group and I don’t know who is who.” Another factor that impeded Moodle and Facebook to form a synergy was due to the different group compositions on two online systems. On Moodle, the students were broken into three smaller groups, whereas on Facebook, there was only one group. Some interviewees also pointed out the problem of having Facebook within formal educational settings: “since some of them (students) have different names on Facebook, I couldn’t link them with the names shown on Moodle.”

Another challenge noted by many students was the insufficient time for Hong Kong and Taiwan students to know each other and cultivate friendship. The online discussion ran for 5 weeks due to the different academic timetables at both places. Some Taiwan students confessed that they were very shy and “slow-to-warm-up”; thus, they became rather passive in such online groups with people they did not know. In this regard, they expressed the need for “knowing each other better” and suggested to “extend time for sharing” and reserve “more time to make new friends.” The students also made other suggestions to enhance sociability such as creating more opportunity for “individual or one-to-one chatting and small group interaction” and “arranging some private Skype chat” with students on the other side.

The other influencing factor concerned the purpose and design of the Facebook group. To begin with, the students felt at a loss as to what to do in such an informal space for social-oriented communications. As one student commented “I do not know the style of conversation.” Furthermore, unlike posts in Moodle forums that would affect their grade, participation in the Facebook group was totally voluntary. For some students, such a design might have caused feelings of the Facebook group as “not serious enough” and decreased their motivation since they were less obliged to participate. Still, some students expressed discomfort to include teachers in the Facebook group. It seems that with teachers’ presence, they found it hard to “get to know each other in a more casual way through discussing less serious topics.” The last factor was associated with the changing practice of the technology. It seems that the students in our study were not that much into Facebook any more as one remarked: “I think Facebook has become outdated in teenagers’ mind.” The other also commented that they did not visit Facebook that frequently any more.

## Discussion and conclusion

In the present study, we attempted to connect students from two geographically separated regions and with different cultural backgrounds via a formal online discussion platform (Moodle) and an informal social-oriented platform (Facebook). In the following discussion, we will review students’ experiences and perceptions of Moodle and Facebook, reflect on the challenges and issues, and followed by suggestions for future practice.

### Online discussion on Moodle

In general, the students at both sides cherished this valuable experience to have course-related online discussions with students from a foreign region. This project was the first-time experience for teachers as well as students at both sides. The feeling of novelty was evident in students’ feedback. They perceived the online discussions on Moodle conducive for exchanging ideas and developing the awareness and appreciation of a different culture. The strength and affordances of online asynchronous discussion was greatly appreciated by our informants. From the instructors’ perspective, one of the most valuable gains from the project is students’ appreciation of the affordances and constraints of digital technologies for connecting, interaction, and learning through first-hand experience. This first experiment of cross-cultural online discussion also yielded valuable insights into the challenges and instructional design issues, which will be elaborated below.

First, both the instructors and students perceived the time limitation as a major obstacle. Admittedly, time is crucial for ice-breaking and establishing social rapport when a new online community is to be formed. In this respect, informal sharing and getting to know each other online could be arranged before the starting point of the semester. Second, language could be a double-edge sword. Language barrier, as reported in many other studies on cross-cultural communication (e.g., Kim & Bonk, 2002; Yang et al., 2014), was also noted in our study. On the positive side, those who were weak in English felt motivated to practice and improve their language ability through online discussion. On the negative side, language barrier deterred the students from joining in the online discussion. To address this, students can be grouped based on their preferred languages, so that they could chat online in the language they are comfortable with. Otherwise, it might be helpful to form English support groups for students who are not so confident with the language, or assign “Chinese language partner” to help those who are not good at communicating in Chinese. Alternatively, it might also be a good idea to recruit “student translator” to help translate the English posts into Chinese or vice versa in order to remove the language barrier.

### Facebook as an informal channel of socialization

Our original design of using Facebook as an informal and social-oriented group space to enhance social presence and interaction was not fully realized. Although many students deemed Facebook appropriate and promising for providing a parallel and complementary platform to Moodle, they felt at a loss how to continue after the initial self-introduction. Reflecting on the reasons behind it, we would like to address four themes emerging from the data: (1) teachers’ presence, (2) group composition, (3) lack of focus, and (4) social relationships and leadership.

First, although the instructors deliberately refrained their presence on Facebook group after setting up the group and suggesting the activities of self-introduction, the initial teachers’ presence, plausibly, gave the students the impression that their interaction there were still under teachers’ radar. This, in a way, affected their outlook of Facebook as a channel informal and casual communication. The same negative attitude towards using Facebook especially teacher-initiated use of Facebook was also reported in Gettman and Cortijo’s (2015) study. Second, another factor that might lead to students’ reluctance in posting on Facebook group might be group composition. Wang (2011) maintained that students in smaller groups showed greater satisfaction with the online interaction via Facebook. Our students also expressed the preferences for small group or even one-on-one interaction online.

Third, when it comes to online community building, several scholars collectively pointed to the importance of a clearly defined focus and purpose (e.g., Salmon, 2000). In our study, we encouraged the students to have casual and free talk on any topic that interested them. Nevertheless, they felt at a loss as to what they should talk about or discuss. Another factor concerns social relationship and leadership that are also important for fostering and sustaining online activities (e.g., Cho, Gay, Davidson, & Ingraffea, 2007). In line with other studies (e.g., Gray, Annabell, & Kennedy, 2010; Thompson, 2007), our students did not know what to talk about in the Facebook group due to the lack of existing social bond or relationships. When the students were faced with unfamiliar people and were left on their own to find a topic for discussion, they felt perplexed. What can we do to address these problems then? We will consider letting the students take the full ownership and responsibility of the informal group on Facebook. One or two active students from Hong Kong and Taiwan will be identified to serve as student leaders. These leaders will take the responsibility of initiating and facilitating social conversation in the online group. In this way, students might feel more comfortable having casual and social chitchat in an online group they could claim their own.

### Create a synergy between formal and informal platforms

Thus far, we have pondered over the lessons learned and insights gleaned on fostering both cognitive and social dimensions of an online group through formal and informal platforms, respectively. A critical question that needs to be addressed is how to synergize both the formal and informal platforms. Facebook, residing primarily in students’ social life, could pose special challenges when introduced into formal learning settings. Dron (2012) found the bottom-up structure of SNS, to some extent, conflicted with the top-down structure of the university courses. In this study, students found it hard to match names shown on Facebook with those on Moodle. The outcome of pooling everyone together within one Facebook group ran counter to our original aim of encouraging more vibrant online interaction and sharing.

The crux of this issue comes down to creating authentic opportunities to link cognitive and social dimensions of learning. In this regard, several students in our study suggested working on group projects in small and culturally mixed groups. Such a design will create a genuine need for students within a group to know each other, communicate through digital technologies, and collaborate with each together. For instance, they could create their own Facebook group or schedule online meetings through Skype. As to the size of group, we would suggest six with three from both sides. Another suggestion, also originally from student feedback, was to have peer review and comments of the group projects. In this design, students will work on group projects with peers from the same university. Each group will be paired with the other group from the foreign university that focuses on similar topics. The two groups will be connected from the outset and engaged in reviewing, commenting, and learning from each other throughout the process.

One of the limitations of the study that we should acknowledge is the different class size of the students on both sides. This made it hard to compare the students on both sides and also posed more pressure on the Taiwan students who were outnumbered by the Hong Kong students. Still, this cross-cultural online discussion experience turned out to be a fruitful and valuable one for both involving teachers and students. The formal online discussion platform engaged students from two regions collectively in reflecting and discussing various issues, which contributed to exchanging perspectives and cross-cultural understanding. On the technological side, the online platform—Moodle—is considered suitable for cross-cultural online discussion. It allows the customization of language for display, thus students from different regions could choose the languages they were comfortable with. For instructors, it is easy to track and monitor students’ online participation and interaction. However, the account settings were quite complicated, thus a skillful technician might be needed to help with creating accounts and resolving technical problems. As to Facebook, although a few students mentioned their subsided interest in Facebook, it is the most popular SNS in both Hong Kong and Taiwan. Although the use of informal Facebook group for socialization had deviated from our expectation, we still think it is a viable option for supplementing formal platform. It is hoped that our reflection and suggestions for future work can benefit others in the similar endeavor.
